# Blood RNA Biomarker Signatures for Early Diagnosis and Prognosis in Ischemic and Hemorrhagic Stroke: The IBIS‐CT1 Study

**DOI:** 10.1002/acn3.70466

**Published:** 2026-07-08

**Authors:** Salomé Retailleau, Marie Bruguet, Irina Viakhireva‐Dovganyuk, François‐Mathias Merrien, Denis Marechal, Aurore Jourdain, Philippe Goas, Jordan Coris, Julien Asselineau, Maëlys Consigny, Gérald Le Gac, Serge Timsit

**Affiliations:** ^1^ Univ Brest, Inserm, EFS, UMR 1078, GGB Brest France; ^2^ Neurology and Stroke Unit Department CHRU de Brest Brest France; ^3^ Clinical Research and Innovation Department CHRU Brest Brest France

**Keywords:** biomarkers, hemorrhagic, ischemic, point‐of‐care, stroke, transcriptomic

## Abstract

**Objective:**

To evaluate the expression of nine blood RNA biomarkers in a clinical trial based on genes previously identified in an experimental monkey model of stroke for diagnosis feasibility and prognostication.

**Methods:**

IBIS‐CT1 was a prospective longitudinal study enrolling patients with ischemic stroke (IS) or intracerebral hemorrhage (ICH) within 6 h of onset, compared with healthy controls (HC). Blood samples were collected at < 6 h (early), 12 h, 24 h, 48 h, 7 days, and 3 months. Target RNAs were quantified using reverse‐transcription PCR or digital droplet PCR. Clinical severity and disability were assessed by NIHSS at admission and the modified Rankin Scale (mRS) at 3 months.

**Results:**

Twenty HC, 20 IS, and 20 ICH patients were included. Compared with HC, IS patients showed significant early upregulation of genes ADM, DUSP1, HMOX1, HSPA1B, LDLR, and PTGS2 (all *p* ≤ 0.0002) and downregulation of BAG3 (*p* < 0.0001). Similar patterns were observed in ICH, with higher expression levels than in IS. In IS, higher NIHSS scores correlated with early upregulation of ADM, DUSP1, HMOX1, PTGS2, and GOS2, while lower BAG3 expression associated with poorer 3‐month outcomes. Combined gene expression discriminated stroke from controls with an AUC of 0.914 (95% CI, 0.84–0.98).

**Interpretation:**

Distinct blood gene‐expression signatures differentiate stroke from controls and predict functional outcome, supporting their potential as diagnostic and prognostic biomarkers.

## 1 Introduction

Major advances in stroke management in recent years have largely relied on optimizing the stroke care pathway across three phases: pre‐hospital, in‐hospital, and post‐hospital. The integration of blood biomarkers at key decision points along this pathway could represent a major breakthrough. (i) Before hospital admission, biomarkers could help distinguish ischemic stroke (IS) from intracerebral hemorrhage (ICH), enabling early targeted treatment—thrombolysis for IS or rapid blood‐pressure control for ICH within mobile stroke units [1, 2, 3]. (ii) In the emergency department, biomarkers could assist in differentiating stroke from stroke mimics, supporting early triage, shortening diagnostic delays, and guiding therapeutic decisions. (iii) After admission, early biomarker profiles could predict long‐term functional outcomes and guide personalized rehabilitation.

Despite a rapidly expanding biomarker field in neurology, no blood biomarker has yet been integrated into routine stroke care. Multiple classes—proteins, nucleic acids, lipids, extracellular vesicles, and metabolites—have been explored. More than 150 candidate protein biomarkers have been investigated for diagnostic or prognostic use [4], yet none have reached clinical implementation [5]. At the acute phase, systematic reviews and meta‐analyses have identified proteins such as BNP, MMP‐9, D‐dimers, and GFAP as potential markers distinguishing IS, ICH, and stroke mimics [6]. GFAP, in particular, has emerged as a promising discriminator of hemorrhagic stroke.

At later stages (≥ 3 months), outcome prediction remains limited, as current prognostic models (e.g., ASTRAL [7]) rely primarily on demographic and clinical variables. A systematic review [8] identified NT‐proBNP, MR‐proANP, copeptin, procalcitonin, mannose‐binding lectin, adipocyte fatty‐acid binding protein, and cortisol as protein biomarkers most consistently associated with poor outcome. However, their incremental predictive value over clinical models remains modest [9].

The use of RNA as a diagnostic biomarker is an emerging field already applied in oncology and infectious diseases. In stroke, blood transcriptomic studies using peripheral blood mononuclear cells or whole blood have investigated mRNA, long non‐coding RNA, extracellular and intracellular microRNA, and circular RNA [10]. These studies aimed to: (i) differentiate IS from controls (often with small cohorts and limited acute‐phase sampling); (ii) distinguish stroke subtypes (large‐vessel, embolic, or lacunar); (iii) identify cryptogenic strokes or transient ischemic attacks; (iv) differentiate ischemic from hemorrhagic stroke [11]; and (v) predict functional outcome [12].

Our preliminary study in a non‐human primate model of middle cerebral artery occlusion [13] identified nine genes differentially expressed both in the brain and in peripheral blood. Eight of these genes—ADM, DUSP1, HMOX1, HSPA1B, LDLR, PTGS2 (COX‐2), G0S2, and BAG3—had previously been implicated in stroke pathophysiology [13], whereas TM4SF1 had not yet been associated with stroke.

The principal objective of the present study (IBIS‐CT1) was to evaluate, in human stroke patients, the expression of these nine RNA biomarkers in blood for diagnostic feasibility, prognostication, and to determine whether their expression is significantly increased in ischemic stroke and hemorrhagic stroke compared with healthy controls and identify their potential in 3‐month prognosis.

## 2 Materials and Methods

### 2.1 Clinical Study

#### 2.1.1 Study Design

IBIS‐CT1 was a prospective longitudinal study with elective groups of patients with ischemic (IS) and intra‐cerebral hemorrhage (ICH) seen before 6 h from stroke onset from November 2020 to May 2023 compared to healthy controls followed‐up to 1 year. Healthy controls (stroke free) were paired to IS patients for age, sex and cardiovascular risk. Hemorrhagic stroke patients were paired to IS patients for age and sex. Data for patients and controls were collected using a standardized case report form (CRF) in a face‐to‐face interview conducted by a neurologist and a clinical research technician (for clinical tests). Controls answered a standardized validated stroke‐free phenotype questionnaire [14], translated into French. Most Controls were recruited from social network and ads in the hospital. A biobank was established at different time after stroke: < 6H from stroke onset, 12 h, 24 h, 48 h, 7 days, 3 months and 1 year from initial sample.

#### 2.1.2 Primary Endpoint

The primary endpoint was the expression (measured in log_2_) of each of the nine targeted genes identified less than 6 h from stroke onset in ischemic and hemorrhagic patients compared to healthy controls.

#### 2.1.3 Secondary Endpoints

The secondary endpoints were:

- The expression (measured in log_2_) of each of the nine‐targeted genes identified at different times from stroke onset after 6 h in ischemic and hemorrhagic patients compared to healthy controls.
- The expression (measured in log_2_) of each nine‐targeted gene identified less than 6 h from stroke onset in ischemic and hemorrhagic patients with good prognosis (Rankin ≤ 2) compared to patients with bad prognosis (Rankin > 2).

#### 2.1.4 Study Population

This monocenter study included patients and controls from the Brest University Hospital (CHRU de Brest).

#### 2.1.5 Inclusion and Exclusion Criteria

For patients, different types of signed consent were used: (i) Procedure of signed consent in situation of emergency; (ii) Consent of the patient if he has the possibility to sign or his representative if he is present. For controls: signed consent. All patients and controls had to have an age > 18 years old.

##### 2.1.5.1 Inclusion Criteria for Ischemic Stroke

Ischemic stroke diagnosed on clinical presentation and cerebral imaging (CT or MRI imaging), inclusion inferior to 6 h from stroke onset, initial NIHSS score > 0 at the time of clinical examination, patients with multimodal imaging either through MRI or CT perfusion and supra‐aortic vessels and intra‐cerebral vessels (Willis circle) imaging < 0.

##### 2.1.5.2 Inclusion Criteria for Intracerebral Hemorrhage

Hemorrhagic stroke diagnosed on clinical presentation and cerebral imaging (CT or MRI imaging), Inclusion inferior to 6 h from stroke onset, initial NIHSS score > 0 at the time of clinical examination. Hemorrhagic patients were paired for age ±5 years and sex with ischemic patients.

##### 2.1.5.3 Inclusion Criteria for Healthy Controls

To be stroke‐free if they answered “no” to all of the questions in the standardized stroke‐free phenotype questionnaire [14], initial NIHSS score = 0, Rankin score = 0, high risk cardiovascular subjects. Controls were paired for age ±2 years, sex and cardiovascular risk measured by a score (European Low Risk Chart) [15] with ischemic patients.

##### 2.1.5.4 Non‐Inclusion Criteria for All

Not affiliated to social security, pregnant or breastfeeding women, patient under legal protection or deprived of liberty by a judicial or administrative decision, patient whose follow‐up will be impossible, prior stroke.

##### 2.1.5.5 Non‐Inclusion Criteria for Ischemic Stroke

Patients with TIA and a negative cerebral CT or MRI.

##### 2.1.5.6 Non‐Inclusion Criteria for Intra‐Cerebral Hemorrhage

Cerebral hemorrhage related to subarachnoid hemorrhage, post‐traumatic hemorrhage, and hemorrhagic transformation in patients with ischemic stroke.

##### 2.1.5.7 Non‐Inclusion Criteria for Healthy Controls

Contraindication to cerebral MRI.

#### 2.1.6 Study Variables

The vascular risk factors identified from medical history were: previous stroke or transient ischemic attack (TIA: lasting < 24 h); carotid surgery; coronary artery disease; cardiac arrhythmia (atrial fibrillation); hypertension; diabetes mellitus; smoking (if daily for > 1 year) or alcohol abuse (if > 3 glasses daily); dyslipidemia; peripheral artery disease; and migraine. Body mass index was calculated for each case with the following categories: Underweight = < 18.5, Normal weight = 18.5–24.9, Overweight = 25–29.9, Obesity = BMI of 30 or greater.

For clinical data, the CRF recorded date of stroke, symptom duration (< 1 h, 1–24 h or > 24 h), weight and height, Glasgow Coma Score (for prospective patients), clinical NIHSS score (on admission for prospective patients and during consultation for healthy controls), and lacunar syndrome. Lacunar syndrome was categorized as pure motor, pure sensory or sensorimotor deficit, dysarthria or clumsy hand, ataxia or hemiparesis, or other. Pre‐ and post‐stroke dependence and disability were assessed on the modified Rankin Scale. It was estimated before stroke and calculated at 3 and 12 months post‐stroke during consultation. Treatment on admission was also recorded, including implementation of thrombolysis and endovascular thrombectomy.

All work‐up data were recorded on the CRF: cerebral CT and MRI, with location of the clinically relevant infarction and its vascular territory; and work‐up to identify stroke mechanism: EKG, cardiac monitoring (24‐h Holter or continuous 24‐h monitoring), echocardiography, extracranial vessel assessment, and intracranial vessel assessment.

#### 2.1.7 Data Validation

A neurologist trained in stroke management validated all patients' status. To validate patient files, neurologists had full access to radiological and hospital data.

#### 2.1.8 Ischemic Stroke Subtypes

For homogeneity, all cases were reviewed by the principal investigator (ST) for TOAST classification [16]. The TOAST classification is based on cardiovascular risk factors, clinical features and diagnostic tests, with 5 categories: large‐artery atherosclerosis (≥ 50% stenosis of a large vessel in absence of other causes); cardioembolism; small‐vessel disease; stroke of other determined cause; and stroke of undetermined cause further subdivided as: 2 or more causes identified, negative assessment (cryptogenic stroke) and incomplete assessment as previously described [17].

#### 2.1.9 Ethics

Identification of Biomarkers in Ischemic Stroke—Clinical Trial (project # 19.12.10.71223 RIPH1 HPS) IBIS‐CT1 trial (https://clinicaltrials.gov/study/NCT04253275) was approved by the Institutional review board approval under the CPP Ile de France 2: n° ID‐RCB/EUDRACT: # 2019‐A03073‐54 and the ANSM (Agence Nationale de Sécurité du Médicament) under the reference #ANSM: HPSAEC1‐2019‐12‐00018. An informed consent was obtained from each subject.

#### 2.1.10 Blood Samples

PAXgene (Quiagen) RNA tubes were maintained in an upright position at room temperature (18°C–25°C) for 2–72 h, followed by incubation at −20°C for 24 h, and subsequently stored at −70°C in a dedicated freezer facility. Samples were stored at the CRB Santé (Biological Resource Center) of Brest The CRB Santé from Brest has engaged in the NF S 96–900 certification (“Management system for a Biological Resource Center and quality of biological resources”), ISO 9001 (“Quality management systems—Requirements”), and ISO 20387 (“Biotechnology—Biobanking—General requirements for biobanking”), and has implemented an organizational structure (quality management system) to ensure the compliance of its biological resources.

#### 2.1.11 Isolation of Total RNA and Reverse Transcription

Total RNA was extracted from patient blood samples with the Maxwell 16 LEV simply RNA Blood Kit according to the manufacturer's instructions (Promega Corporation). Briefly, blood samples were centrifuged 10 min at 3000*g* and the supernatant discarded. After a washing step, white blood cells were mixed with 1‐Thioglycerol/homogenization solution and then with Lysis Buffer and Proteinase K. Finally, after cartridges preparation, samples were loaded and run on the Maxwell 16 Instrument for an automated RNA purification. RNA samples were eluted in 50 μL Nuclease‐free water. Three independent reverse transcriptions were performed for each RNA samples. gDNA was removed from RNA samples using DNase I Amp Grade (ThermoFisher Scientific). cDNA were generated from 125 ng total RNA, using 2,5 μM anchored Oligo dT22 (Eurogentec), 500 μM of dNTP (Ozyme) and 200 U of SuperScript III reverse transcriptase (ThermoFisher Scientific) according to the manufacturer's protocol. cDNA were finally diluted by 1.5.

#### 2.1.12 Primers and Probes

Primers and probes used in this study are listed in Table S1. All primers and probes were synthesized by Eurogentec. BAG3, G0s2 and TM4SF1 probes were labeled with the 6‐carboxyfluorescein (FAM) molecule at the 5′‐end and with the quencher MGB‐Eclipse at the 3′‐end. PPP6R3 probe was labeled with hexachloro‐fluorescein (HEX) molecule at the 5′‐end and with the quencher MGB‐Eclipse at the 3′‐end. Forward and reverse primers were designed to target different exons of genes in order to amplify only mRNA transcripts.

#### 2.1.13 qPCR Assay

Target RNAs were monitored using a quantitative PCR approach (fluorescent nucleic acid staining strategy). The real‐time PCR efficiency (E) of each of the target and reference gene transcripts was previously studied using geometric dilutions of cDNA. Relative expression ratios were then calculated using the mathematical model described by Pfaffl [18]. Each RNA sample was examined in three independent experiments as previously described [13]. $\text{Fold change}=\frac{E_{\text{Target}}^{\left({\mathrm{CT}}_{\text{Control Target}}-{\mathrm{CT}}_{\text{Sample Target}}\right)}}{E_{\mathrm{Ref}}^{\left({\mathrm{CT}}_{\text{Control}\;\mathrm{Ref}}-{\mathrm{CT}}_{\text{Sample}\;\mathrm{Ref}}\right)}}$. CT: cycle threshold; Control/Sample: control (no stroke) or patient (ischemic or hemorrhagic); Target/Ref: targeted gene or reference gene were RPL13A as previously described [19] or SMC2 as previously identified in monkey [13]. Data were shown as log_2_ (Fold Change).

The quantitative real‐time PCR reactions were run on the Light Cycler 480 Instrument II (Roche Diagnostics). Thermocycling was performed in a final volume of 20 μL containing 1× HotStar Taq Master Mix (Qiagen), 500 nM of each forward and reverse primer (Table S1), 500 nM of Syto 9 green fluorescent nucleic acid stain (ThermoFisher Scientific), and 2 μL of diluted cDNA. The following program was used for amplification: enzyme activation at 95°C for 15 min, 40 cycles consisting of 15 s of denaturation at 95°C, 30 s of hybridization at 59°C, and 30 s of extension at 72°C. Melting curves were inspected for single peaks, and PCR products were subjected to agarose gel electrophoresis to ensure specific amplification of the products of interest.

#### 2.1.14 ddPCR Assay

The digital droplet PCR reactions were prepared and run on the QX200 Droplet Digital PCR (ddPCR) System following the manufacturer's instructions. Thermocycling was performed in a final volume of 40 μL of droplets containing 1× ddPCR Supermix for probes (No dUTP) (Bio‐RAD), 900 nM of each forward and reverse primer, 270 nM of FAM‐based (target gene) and HEX‐based (housekeeping gene) probes, and 1 μL of diluted cDNA. The following program was used for amplification: enzyme activation at 95°C for 10 min, 40 cycles consisted of 30 s of denaturation at 94°C and 1 min of hybridization/extension at 58°C, and enzyme deactivation at 98°C for 10 min. The data analysis was performed with QuantaSoft droplet reader software (Bio‐RAD). All ddPCR data were normalized with the PPP6R3 gene, identified by RNA sequencing (data not shown) as expressed in all samples, stable (SD < 0.2 from log_2_ values), and mapping a single location in the genome (> 95% of uniquely mapped reads). RPL13A was not suitable as a normalization gene because of its high expression in samples.

#### 2.1.15 Statistical Analysis

– Baseline characteristics are described by group (ischemic, hemorrhagic and control) with frequency and percentages for qualitative variables, median and quartile for quantitative variables.
– For analysis of mRNA expression, data were expressed as the mean ± standard deviation (SD).
– One sample *t*‐test or Wilcoxon signed‐rank test was used to compare IS or ICH versus healthy patients on the log_2_ (fold change).
– Paired *t*‐test or paired Wilcoxon signed‐rank test was used to compare the log_2_ (fold change) between IS and ICH.
– Two sample *t*‐tests or Wilcoxon signed rank tests were used to compare the log_2_ (fold change) between clinical subgroups (NIHSS, Glasgow, RANKIN)
– For the estimation of the potential of the 9 genes' expression or their combination to predict a stroke (versus no‐stroke), area under the ROC curve (AUC), sensitivity, and specificity (after having determined a threshold to obtain a sensitivity of at least 90%) were calculated based on the number of PCR cycles normalized with a reference gene. A logistic regression model without any selection approach was used to estimate the predictive accuracy of the combination of the 9 genes' expression.
– For correlation of mRNA expression with clinical severity and 3‐month handicap, Pearson correlation or Spearman rank correlation was applied depending on normality distribution.

Due to multiple comparisons, statistical significance was set at 0.01 for comparison between genes expression and at 0.05 for other analysis. Statistical analysis was performed using SAS software version 9.4 and Graph Pad Prism 5 software.

## 3 Results

### 3.1 Patient's Baseline Characteristics and Handicap at Three Month (Table 1)

**TABLE 1:** Patient's baseline characteristics and handicap at 3 months.

Characteristics	Ischemic stroke (*n* = 20)	Intra‐cerebral hemorrhage (*n* = 20)	Healthy controls (*n* = 20)
Median; Q1–Q3			
*N*, %			
Age	76.5 (66.5; 82.0)	74.5 (66.5; 82.5)	75.5 (66.0; 79.5)
Female	12 (60.0%)	12 (60.0%)	12 (60.0%)
Euroscore	2.5 (1.5; 4.0)	2.0 (2.0; 4.0)	2.5 (2.0; 4.0)
Clinical parameters			
Median Rankin before stroke	0.0 (0.0; 1.0)	0.0 (0.0; 2.5)	0.0 (0.0; 0.0)
Median NIHSS	16.0 (7.5; 19.5)	14.0 (8.5; 18.5)	0.0 (0.0; 0.0)
Median GCS	15.0 (12.0; 15.0)	15.0 (13.0; 15.0)	15.0 (15.0; 15.0)
CHADS score	4.0 (3.0; 7.0)	4.0 (3.0; 4.0)	3.0 (3.0; 3.0)
Coronary heart disease	2 (10.0%)	1 (5.0%)	1 (5.0%)
Atrial fibrillation	3 (15.0%)	5 (25.0%)	1 (5.0%)
Lower limb arteriopathy	2 (10.0%)	1 (5.0%)	0 (0.0%)
Hypertension	11 (55.0%)	10 (50.0%)	10 (50.0%)
Diabetes mellitus	1 (5.0%)	3 (15.0%)	0 (0.0%)
Dyslipidemia	7 (35.0%)	5 (25.0%)	9 (47.4%)
Smoking status			
Old	6 (33.3%)	6 (35.3%)	6 (30.0%)
Current	4 (22.2%)	0 (0.0%)	3 (15.0%)
High alcohol intake			
Old	1 (5.6%)	0 (0.0%)	0 (0.0%)
Current	1 (5.6%)	2 (11.8%)	0 (0.0%)
Clinical handicap at 3 months			
Rankin score	3.0 (2.0; 5.0)	5.0 (1.5; 6.0)	0.0 (0.0; 0.0)

Sixty‐one patients were included in the study but one patient was excluded from the ischemic stroke group because stroke was not confirmed. Therefore, 20 healthy controls were studied, 20 ischemic stroke, and 20 intracerebral hemorrhage.

Median age was 76.5 for ischemic stroke patients, 75.5 for healthy controls, and 74.5 for intracerebral hemorrhage patients. Sixty percent were female in all three groups, and median Euroscore was 2.5 for ischemic stroke patients and healthy controls and 2.0 for ICH patients. No clinical difference was observed for demographic variables and Euroscore. Median NIHSS was 16 for IS, 14 for ICH, and 0 for healthy controls. CHADS score was not different among groups. About 50% or more had hypertension whether ischemic, hemorrhagic, or controls. Clinical handicap was more severe in ICH patients (median Rankin score = 5) compared to ischemic stroke (median Rankin score = 3).

### 3.2 Blood RNA Expression

Nine genes were identified in blood of primates [13] but only 6 could be detected by quantitative RT‐PCR (ADM, DUSP1, HMOX1, HSPA1B, LDLR, and PTGS2); the 3 remaining genes (BAG3, G0S2, and TM4SF1) were hardly detected by RT‐qPCR and were therefore analyzed by droplet ddPCR (ddPCR).

### 3.3 Whole Blood mRNA Expression by RT‐qPCR of 6 Identified Genes in Ischemic and Hemorrhagic Stroke Across Time Since Stroke Onset With Two Housekeeping Genes: RPL13A and SMC2

**FIGURE 1:**
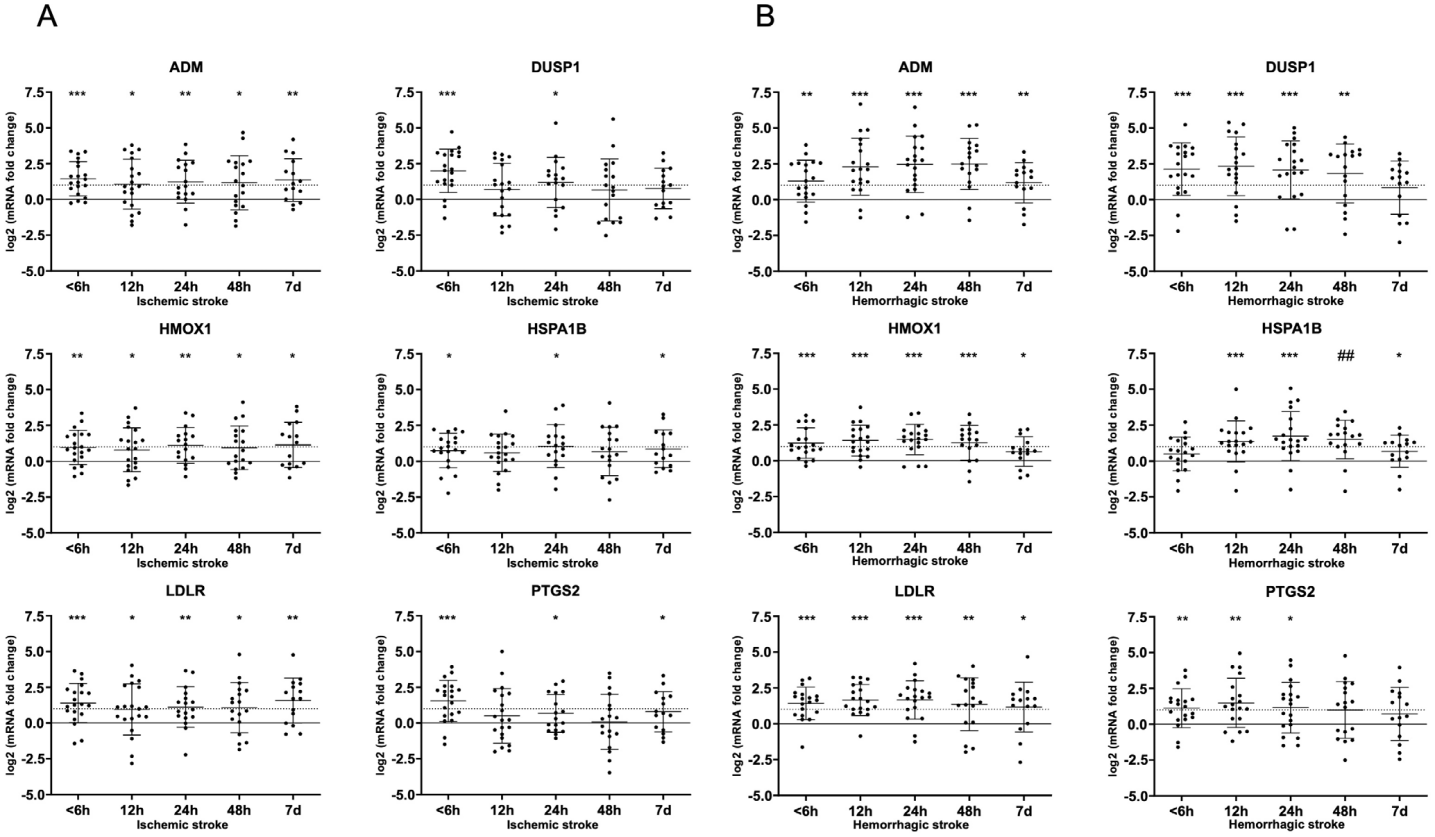
Time course of mRNA relative expression of 6 identified genes in ischemic stroke and hemorrhagic stroke. (A) Ischemic stroke. Determination by RT‐qPCR of the relative expression of ADM, DUSP1, HMOX1, HSPA1B, LDLR and PTGS2 at admission (< 6 h), 12 h, 24 h, 48 h and 7 days after symptoms onset in the whole blood of 20 ischemic patients compared to 20 paired controls. Gene expression levels were normalized to the housekeeping gene RPL13A. One sample *t*‐test: *p ≤ 0.05; **p ≤ 0.01; ***p ≤ 0.001. (B) Hemorrhagic stroke. Determination by RT‐qPCR of the relative expression of ADM, DUSP1, HMOX1, HSPA1B, LDLR and PTGS2 at admission (< 6 h), 12 h, 24 h, 48 h and 7 days after symptoms onset in the whole blood of 20 hemorrhagic patients compared to 20 paired controls. Gene expression levels were normalized to the housekeeping gene RPL13A. One sample *t*‐test: *p ≤ 0.05; **p ≤ 0.01; ***p ≤ 0.001. Wilcoxon signed‐rank test: ^##^ *p* ≤ 0.01.

Quantitative PCR was used to measure the relative expression of ADM, DUSP1, HMOX1, HSPA1B, LDLR, and PTGS2 (Figure 1) at different times after stroke onset.

#### 3.3.1 Ischemic Stroke (IS)

On admission for ischemic stroke (Figure 1A), the level of all mRNA genes with RPL13A housekeeping gene was increased compared to paired controls for ADM (log_2_ (FC) = 1.44, *p* = 0.0001), DUSP1 (log_2_ (FC) = 2.00, *p* = 0.0001), HMOX1 (log_2_ (FC) = 0.97, *p* = 0.0016), HSPA1B (log_2_ (FC) = 0.75, *p* = 0.0118), LDLR (log_2_ (FC) = 1.40, *p* = 0.0002), and PTGS2 (log_2_ (FC) = 1.54, *p* = 0.0001) (Table S2). After admission, with RPL13A normalization, at 24 H, ADM, HMOX1, and LDLR mRNA, and at 7 days, ADM and LDLR, were overexpressed (*p* < 0.01) compared to paired controls. Their mean expression was quite stable upon 7 days post‐IS.

To confirm our results another housekeeping gene, SMC2 (Figure S3A) was used. Similar results were obtained with a mRNA level increase at admission of ADM (log_2_ (FC) = 1.00, *p* = 0.0031), DUSP1 (log_2_ (FC) = 1.55, *p* = 0.0002), HMOX1 (log_2_ (FC) = 0.52, *p* = 0.0161), LDLR (log_2_ (FC) = 0.91, *p* = 0.0012) and PTGS2 (log_2_ (FC) = 1.09, *p* = 0.0010) when compared to paired controls (Table S2). In addition, HMOX1 and LDLR mRNA levels were respectively significantly increased at 24H and 7 days post‐IS in comparison with mRNA levels from healthy paired controls (Figure S3A).

#### 3.3.2 Hemorrhagic Stroke (ICH)

On admission for hemorrhagic stroke (Figure 1B), 5 out of the 6 studied genes were significantly overexpressed with RPL13A housekeeping gene for ADM (log_2_ (FC) = 1.29, *p* = 0.0012), DUSP1 (log_2_ (FC) = 2.13, *p* = 0.0001), HMOX1 (log_2_ (FC) = 1.24, *p* = 0.0001), LDLR (log_2_ (FC) = 1.43, *p* = 0.0001), and PTGS2 (log_2_ (FC) = 1.13, *p* = 0.002) (Table S2). After admission, with RPL13A normalization, ADM (12H, 24H, 48H, 7D), DUSP1 (12H, 24H, 48H), HMOX1 (12H, 24H, 48H), HSPA1B (12H, 24H, 48H), LDLR (12H, 24H, 48H), and PTGS2 (12H) were overexpressed (*p* ≤ 0.01) when compared to paired controls with a peak at 24 h post‐ICH for ADM, HMOX1, HSPA1B while DUSP1 and PTGS2 genes showed an earlier expression peak at 12 h post‐ICH (*p* ≤ 0.01) with a progressive decline from 12 h to 7 days post‐ICH.

Using SMC2 housekeeping gene for qPCR normalization led to similar observations (Figure S3B): ADM (log_2_ (FC) = 0.91, *p* = 0.0298), DUSP1 (log_2_ (FC) = 1.74, *p* = 0.0013), HMOX1 (log_2_ (FC) = 0.85, *p* = 0.0011), LDLR (log_2_ (FC) = 1.07, *p* = 0.0027) and PTGS2 (log_2_ (FC) = 0.77, *p* = 0.0364) (Table S2) mRNA levels were increased in the blood of hemorrhagic patients on admission when compared to healthy paired controls. After admission, with SMC2 normalization, ADM (12H, 24H, 48H), DUSP1 (12H, 24H), HMOX1 (12H, 24H), LDLR (12H, 24H) were overexpressed (*p* ≤ 0.01) when compared to paired controls. Similarly, to results obtained from RPL13A normalization, DUSP1 reached a peak of expression at 12 h post‐ICH with a gradual reduction until 7 days (Figure S3B).

#### 3.3.3 Comparison of mRNA Expression Between IS and ICH Across Time

**FIGURE 2:**
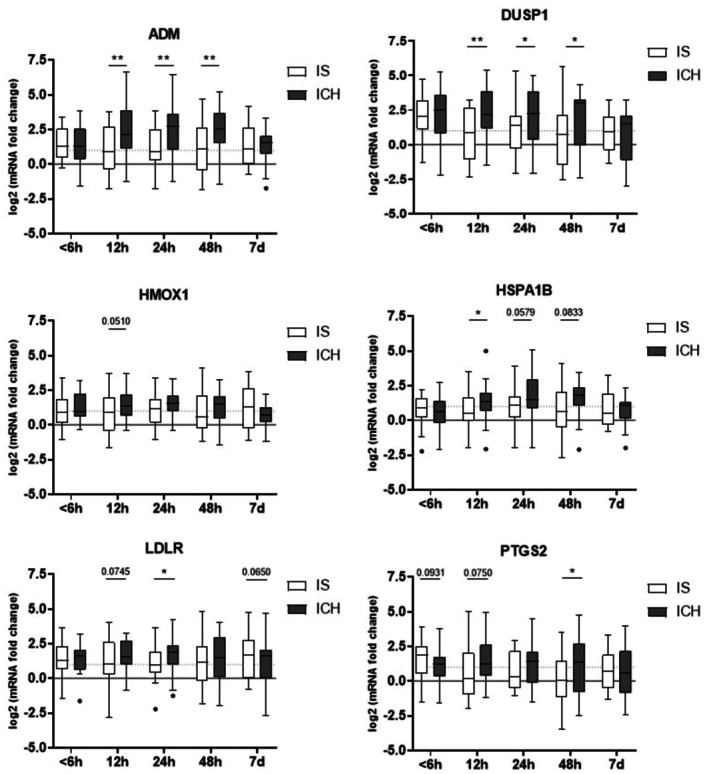
Comparative time course of mRNA relative expression of 6 identified genes in ischemic and hemorrhagic stroke. Determination by RT‐qPCR of the relative expression of ADM, DUSP1, HMOX1, HSPA1B, LDLR, and PTGS2 at admission (< 6 h), 12 h, 24 h, 48 h, and 7 days after symptoms onset in the whole blood of 20 ischemic patients compared to 20 paired hemorrhagic patients. Gene expression levels were normalized to the housekeeping gene RPL13A. Paired *t*‐test: *p ≤ 0.05; **p ≤ 0.01.

Figure 2 showed mRNA expression of IS and ICH across time compared to paired controls. No significant different relative expression was observed for the six studied genes between IS and ICH at patient admission. mRNA levels for ADM were significantly higher at 12H, 24H, and 48H and DUSP1 at 12H, in ICH patients compared to IS. Likewise, HSPA1B, LDLR, and PTGS2 showed no significant greater mRNA expression at 12 h, 24 h, and 48 h post‐stroke respectively in the ICH cohort compared to IS. Normalization by SMC2 confirmed our first observations made for ADM at 24H and DUSP1 at 12H mRNA (Figure S4). Figure S2 showed mRNA expression kinetic curves of IS and ICH. IS and ICH had clearly different profiles. Several genes (ADM, DUSP1, HSPA1B, and PTGS2) exhibited early and higher expression in hemorrhagic stroke compared with ischemic stroke, whereas ischemic stroke was characterized by more stable or progressively increasing expression patterns over time.

### 3.4 Expression on Admission of 6 Identified Genes by RT‐qPCR According to Stroke Severity in IS and ICH With 2 Housekeeping Genes

**FIGURE 3:**
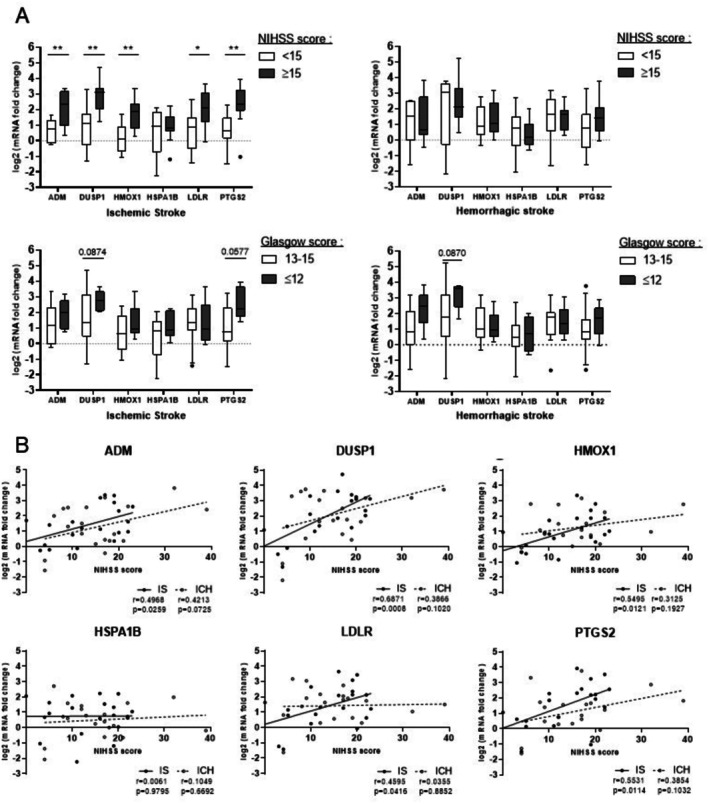
Relative mRNA expression of 6 identified genes according to initial clinical severity in ischemic and hemorrhagic stroke. (A) Stratification of ADM, DUSP1, HMOX1, HSPA1B, LDLR and PTGS2 mRNA relative expression according to NIHSS score at admission (< 15, ≥ 15) in the whole blood of ischemic patients (IS) (*n* = 20) and hemorrhagic patients (ICH) (*n* = 19) and Glasgow coma scale (GCS) (≤ 12, > 12) at admission in the whole blood of ischemic patients (*n* = 19) and hemorrhagic patients (*n* = 19). For NIHSS, impaired *t*‐test: *p ≤ 0.05; **p ≤ 0.01 and for GCS Mann–Whitney test. (B) Pearson correlation of genes relative expression with NIHSS score at admission (filled dot = IS patients; empty dot = ICH patients). Note significant correlation in IS for ADM, (*p* = 0.026); DUSP1 (*p* = 0.001), HMOX1 (*p* = 0.012), LDLR (*p* = 0.042) and PTGS2 (*p* = 0.011). No significant correlation for ICH.

In each cohort, patients were divided in 2 groups according to the severity of stroke, measured with the NIHSS score and the Glasgow coma scale (GCS) (Figure 3). Patients with a NIHSS score below 15 were classified as mild to moderately severe stroke, while NIHSS scores ≥ 15 were classified as severe to very severe stroke [20]. In the same manner, a GCS score of 13 or greater suggested a mild brain injury, while a GCS score below 12 indicated moderate to severe brain injury [21].

– *For the ischemic stroke cohort* (Figure 3), ADM, DUSP1, HMOX1 and PTGS2 genes showed a higher mRNA relative expression in the group of high NIHSS score in comparison to the group of low NIHSS score (Unpaired *t*‐test *p* ≤ 0.01) (Figure 3A). The correlation analysis (Figure 3B) showed that 5 genes were positively correlated with NIHSS score on admission for ADM (*r* = 0.50, *p* = 0.026), DUSP1 (*r* = 0.69, *p* = 0.001), HMOX1 (*r* = 0.55, *p* = 0.012), LDLR (*r* = 0.46, *p* = 0.042) and PTGS2 (*r* = 0.55, *p* = 0.011). DUSP1 gene had the strongest correlation factor with NIHSS score. HSPA1B mRNA relative expression was not correlated with the NIHSS score. Regarding the GCS no difference was observed (Figure 3A).

When using SMC2 housekeeping gene for normalization, higher and significant gene expression (*p* < 0.01) for HMOX1 (Figure S5A) was observed when comparing NIHSS severity patients. ADM (*p* = 0.045), HMOX1 (*p* = 0.024), and PTGS2 (*p* = 0.043) were positively correlated with NIHSS score on admission (Figure S5B).

– *For the hemorrhagic stroke cohort* (Figure 3), the mRNA relative expression was not significantly different between the two NIHSS groups (Figure 3A). No correlation with NIHSS was observed for HMOX1, HSPA1B, and LDLR genes. As for the GCS (Figure 3A), no significant difference was observed.

When using SMC2 housekeeping gene (Figure S5A) for normalization, still none of the genes showed significant and different expression between the two clinical severity NIHSS scores. Regarding the correlation analysis (Figure S5B), no significant correlation was observed.

#### 3.4.1 Expression on Admission of 6 Identified Genes by RT‐qPCR According to 3‐Months Clinical Handicap in IS and ICH

**FIGURE 4:**
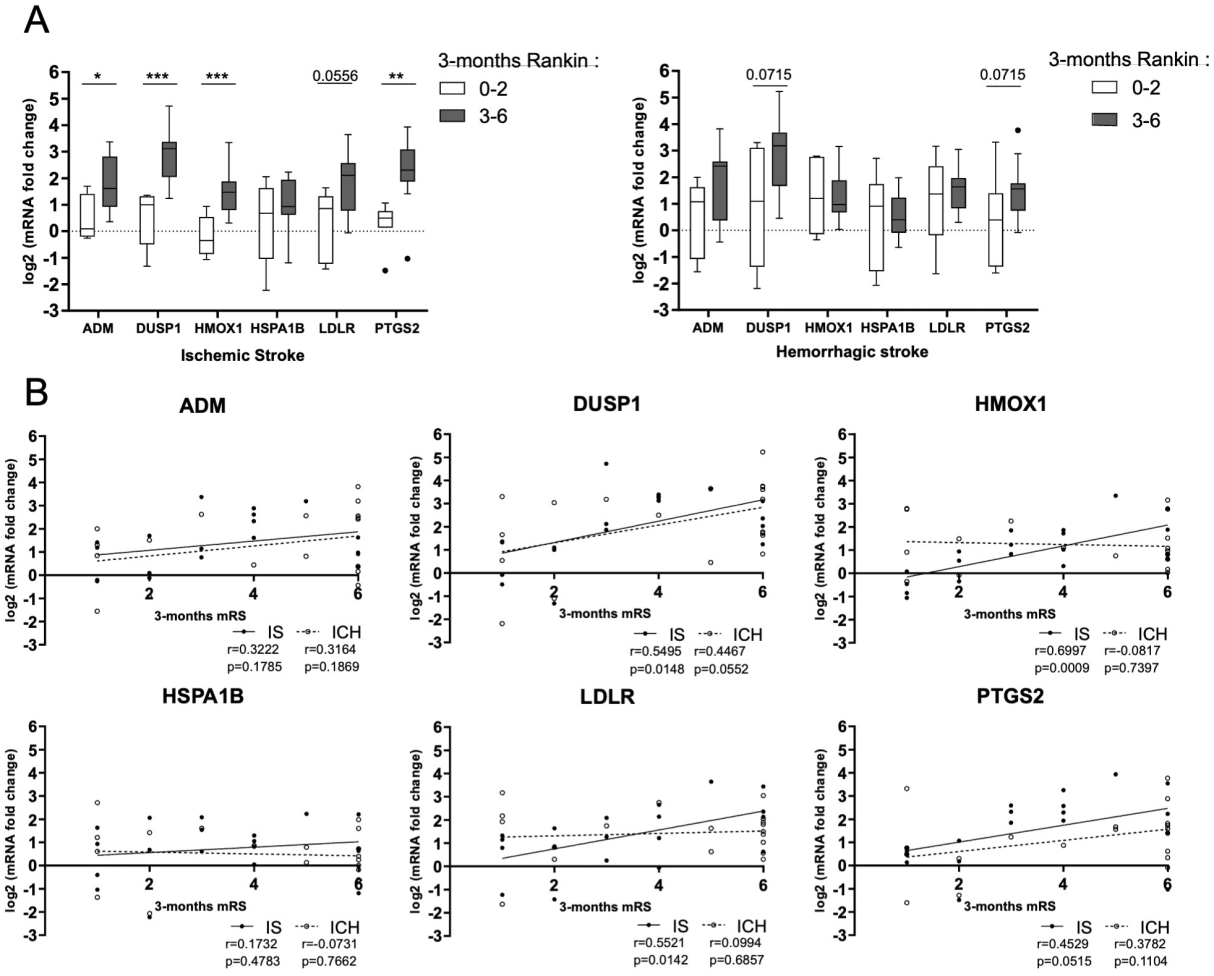
Relative mRNA expression of 6 identified genes according to 3‐months clinical handicap in ischemic and hemorrhagic stroke. (A) Stratification of ADM, DUSP1, HMOX1, HSPA1B, LDLR, PTGS2 mRNA relative expression according to 3‐months Rankin score (≤ 2, > 2) from symptoms onset in ischemic stroke patients (IS) (*n* = 19) and hemorrhagic patients (ICH) (*n* = 19). Mann–Whitney test: *p ≤ 0.05; **p ≤ 0.01; ***p ≤ 0.001. (B) Pearson correlation of genes relative expression with 3‐months Rankin score (filled dots = IS patients; empty dots = ICH patients). Note significant correlation in IS for DUSP1 (*p* = 0.015), HMOX1 (*p* = 0.0009), LDLR (*p* = 0.014) and trend for PTGS2 (*p* = 0.052). For ICH trend for DUSP1 (*p* = 0.055).

The patient clinical handicap was assessed using the modified Rankin score (mRS) and measured 3 months after symptoms onset (Figure 4). Commonly, a mRS below or equal to 2 [22] is considered as no or slight disability, whereas a higher score is associated with moderate to severe disability.

*For the ischemic stroke cohort*, ADM (*p* = 0.036), DUSP1 (*p* = 0.0002), HMOX1 (*p* = 0.001), PTGS2 (*p* = 0.001) showed a higher mRNA relative expression in the group with severe handicap in comparison with patients with lower Rankin (Figure 4A). Correlation analysis (Figure 4B) showed a significant correlation for HMOX1 (*r* = 0.70; *p* = 0.001) and for DUSP1 (*r* = 0.55, *p* = 0.015), LDLR (*r* = 0.55; *p* = 0.014) genes expression.

When using SMC2 housekeeping gene (Figure S6), ADM did not show significantly higher mRNA expression in the high mRS group compared to the lower mRS group. Only DUSP1, and HMOX1 genes exhibited a significant gene expression difference between the two mRS groups, the mRNA levels being greater in the severe 3‐months disability group. As with RPL13A normalization, HMOX1 gene expression was positively and significantly correlated with 3‐month mRS (Figure S6B).

*For the hemorrhagic patient's cohort*, no significant associations were obtained between gene expression and mRS (Figure 4).

When normalizing results with SMC2 (Figure S6), ICH patients from the 3–6 mRS group exhibited higher ADM, DUSP1, and PTGS2 gene expression in comparison to the lower mRS group (Figure S6A). The correlation analysis (Figure S6B) reflected these same observations: ADM (*r* = 0.48, *p* = 0.037), DUSP1 (*r* = 0.58, *p* = 0.001), and PTGS2 (*r* = 0.58, *p* = 0.001) gene expression significantly and positively correlated with 3‐month mRS.

### 3.5 Whole Blood mRNA Expression by ddPCR of 3 Identified Genes in Ischemic and Hemorrhagic Stroke on Admission

**FIGURE 5:**
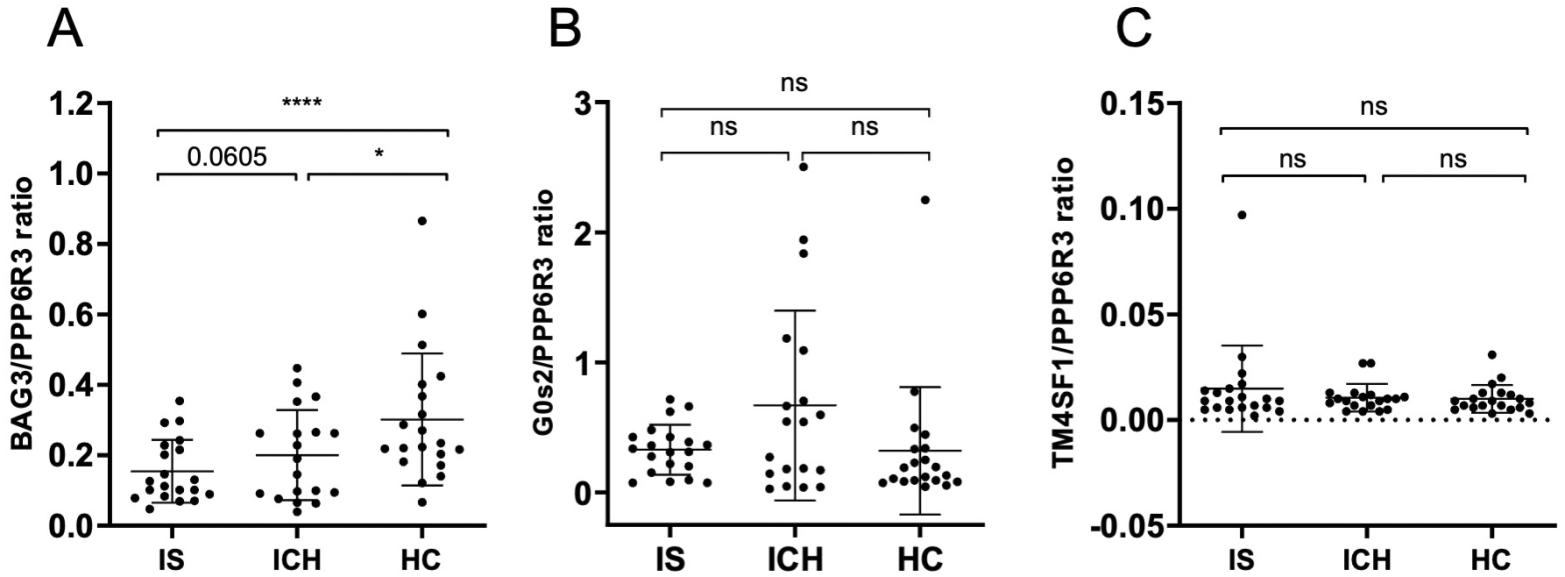
mRNA expression of 3 identified genes in ischemic and hemorrhagic stroke on admission Determination by RT‐ddPCR of the absolute expression of BAG3, G0S2 and TM4SF1 at admission (< 6 h) in the whole blood of 20 ischemic stroke patients (IS), 19 hemorrhagic stroke patients (ICH) and 20 paired healthy controls (HC). Gene expression levels were normalized to the housekeeping gene PPP6R3. Wilcoxon matched‐pairs signed rank test: *p ≤ 0.05; ****p ≤ 0.0001.

ddPCR was used to measure the absolute mRNA expression of BAG3, G0s2 and TM4SF1 (Figure 5).

Figure 5 showed a significantly greater expression of BAG3 in controls compared to both IS (*p* < 0.0001) and ICH (*p* = 0.032) patients. In addition, BAG3 mRNA level was non‐significantly higher (*p* = 0.060) in the ICH compared to the IS cohort. However, the expression of BAG3, G0S2, and TM4SF1 mRNA levels was not significantly different between the healthy controls, the IS, and ICH.

### 3.6 Expression on Admission of 3 Identified Genes by ddPCR According to Stroke Severity in IS and ICH

**FIGURE 6:**
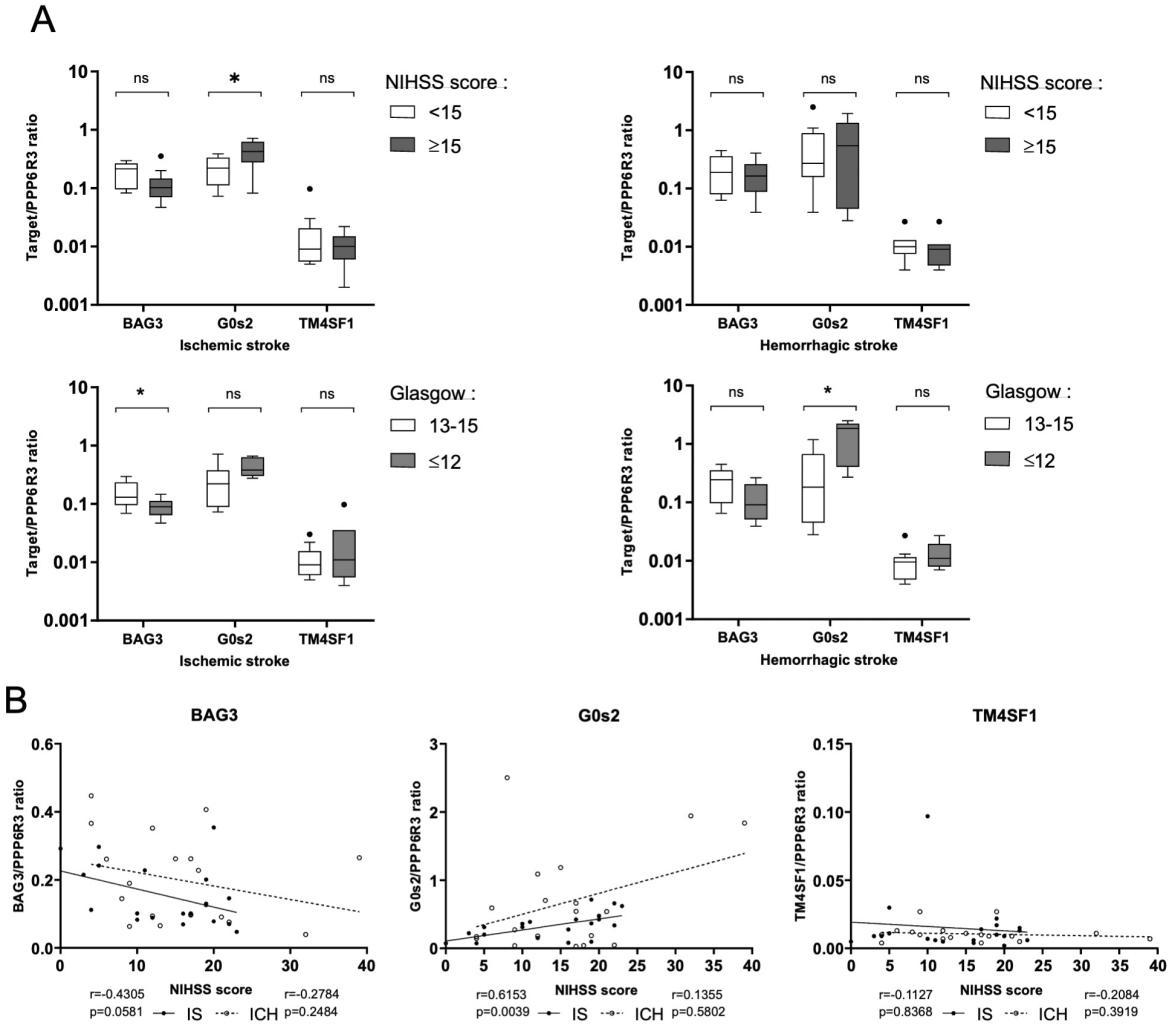
mRNA expression of 3 identified genes according to initial clinical severity in ischemic and hemorrhagic stroke. (A) Stratification of BAG3, G0s2 and TM4SF1 absolute mRNA expression according to NIHSS score (< 15, ≥ 15) and Glasgow Coma Scale (GCS) (≤ 12, > 12) at admission in the whole blood of 20 ischemic patients and 19 hemorrhagic patients. Unpaired *t*‐test or Mann–Whitney test: *p ≤ 0.05. (B) Correlation of mRNA genes absolute expression with NIHSS score at admission (filled dots = IS patients; empty dots = ICH patients).

– For ischemic cohort, no significant difference was observed. Correlation analysis (Figure 6B) showed that G0S2 in IS patients was positively correlated (*r* = 0.62; *p* = 0.004) with NIHSS score. Regarding the GCS, no significant difference was observed (Figure 6A).
– For hemorrhagic cohort, no difference in NIHSS score severity was observed for BAG3, G0S2 and TM4SF1 (Figure 6A). No correlation was observed between the 3 genes expression level and NIHSS score (Figure 6A). For GCS no difference was observed (Figure 6B).

### 3.7 Expression on Admission of 3 Identified Genes by ddPCR According to 3‐Months Clinical Handicap in IS and ICH

**FIGURE 7:**
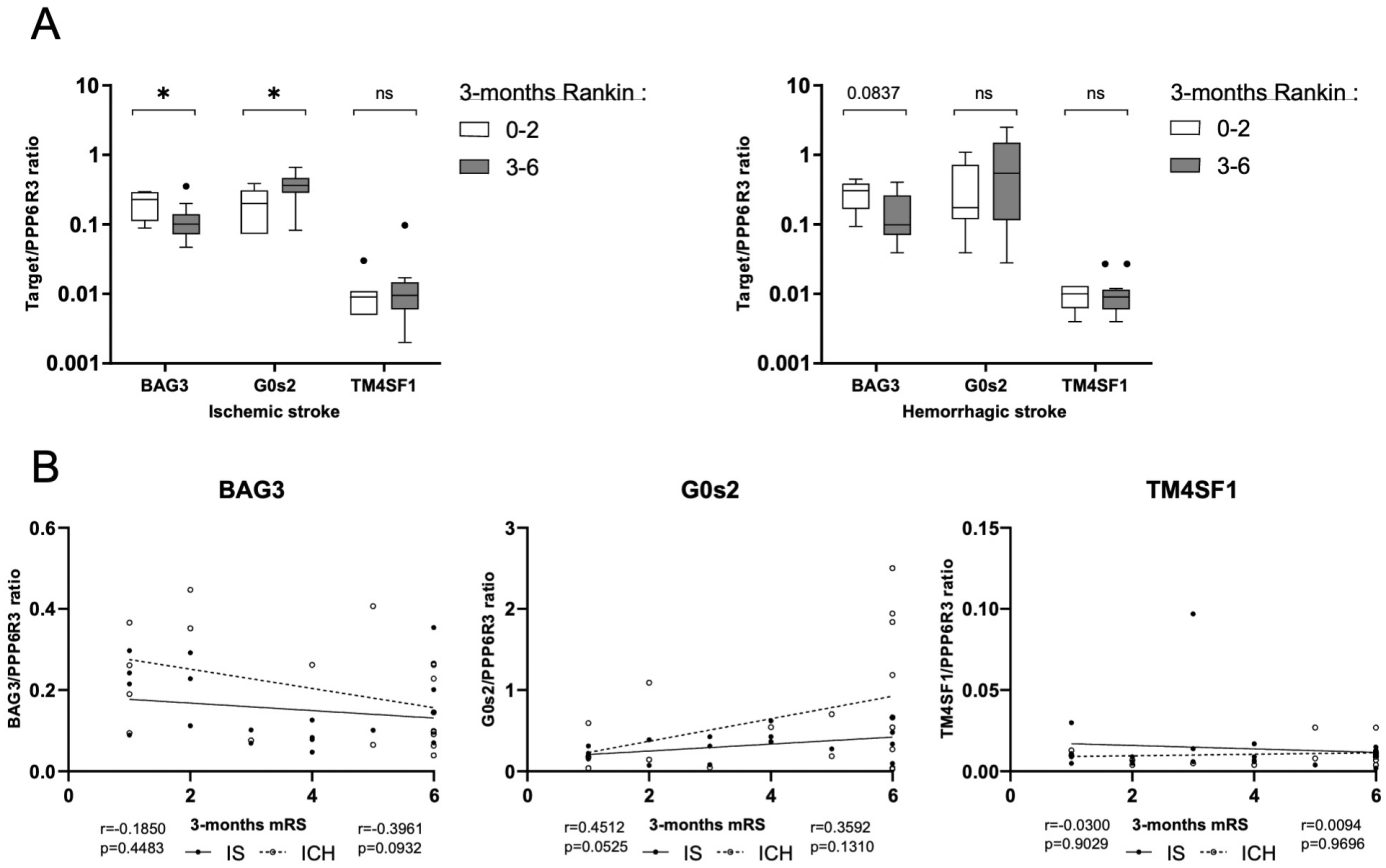
mRNA expression of 3 identified genes according to 3‐month clinical handicap in ischemic and hemorrhagic stroke. (A) Stratification of BAG3, G0s2 and TM4SF1 absolute mRNA expression according to 3‐months Rankin score (≤ 2, > 2) from symptoms onset in ischemic stroke patients (IS) (*n* = 19) and hemorrhagic patients (ICH) (*n* = 19). Mann–Whitney test: *p ≤ 0.05. (B) Correlation of genes absolute expression with 3‐months Rankin score (filled dots = IS patients; empty dots = ICH patients).

– For the ischemic stroke cohort (Figure 7), no significant difference was observed for BAG3, GOS2 and TM4SF1 (Figure 7A,B).
– For the hemorrhagic cohort (Figure 7), BAG3 mRNA expression was non‐significantly lower (*p* = 0.084) in the severe handicap group compared to the mild handicap patients (Figure 7A). No difference for G0S2 and TM4SF1 mRNA expression was observed. This result was consistent with BAG3, G0S2, and TM4SF1 mRNA expression not being correlated to 3‐month mRS (Figure 7B) correlated with 3‐month Rankin score (Figure 7B).

### 3.8 Predictive Accuracy of the Combination of 9 RNA Expression to Diagnose a Stroke Versus No‐Stroke

Table 2 shows the accuracy of a test based on mRNA expression of 9 genes before 6H from stroke onset either used individually or in combination for the diagnostic of stroke (Ischemic and hemorrhagic) versus no‐stroke. Combination of genes shows that AUC could be much higher (AUC = 0.914; 95% CI: 0.84–0.98) than for individual genes prediction and more specific.

**TABLE 2:** Potential accuracy of an early test (< 6H) for the diagnosis of stroke versus no‐stroke.

Genes	AUC [95% CI]	Positivity threshold	Sensitivity [95% CI]	Specificity [95% CI]
Normalization by RPL13A				
HSPA1B	0.628 [0.478–0.779]	1138.535	92.3 [79.1–98.4]	10.0 [1.23–31.7]
PTGS2	0.764 [0.634–0.895]	4570.784	92.3 [79.1–98.4]	25.0 [8.66–49.1]
ADM	0.788 [0.674–0.903]	121.013	92.3 [79.1–98.4]	35.0 [15.4–59.2]
DUSP1	0.864 [0.775–0.954]	21.051	92.3 [79.1–98.4]	45.0 [23.1–68.5]
HMOX1	0.746 [0.616–0.877]	3064.658	92.3 [79.1–98.4]	35.0 [15.4–59.2]
LDLR	0.779 [0.646–0.912]	40842.32	92.3 [79.1–98.4]	50.0 [27.2–72.8]
Normalization by SMC2				
HSPA1B	0.555 [0.397–0.714]	2.737	92.3 [79.1–98.4]	10.0 [1.23–31.7]
PTGS2	0.705 [0.573–0.838]	10.397	92.3 [79.1–98.4]	25.0 [8.66–49.1]
ADM	0.712 [0.576–0.847]	0.358	92.3 [79.1–98.4]	30.0 [11.9–54.3]
DUSP1	0.795 [0.683–0.907]	0.049	92.3 [79.1–98.4]	25.0 [8.66–49.1]
HMOX1	0.672 [0.526–0.817]	11.273	92.3 [79.1–98.4]	20.0 [5.73–43.7]
LDLR	0.710 [0.571–0.849]	135.95	92.3 [79.1–98.4]	25.0 [8.66–49.1]
Digital PCR				
BAG3	0.690 [0.531–0.848]	9.844	92.3 [79.1–98.4]	40.0 [19.1–63.9]
G0S2	0.684 [0.533–0.835]	9.116	92.3 [79.1–98.4]	30.0 [11.9–54.3]
TM4SF1	0.674 [0.517–0.832]	8.728	92.3 [79.1–98.4]	30.0 [11.9–54.3]
Combination (normalization by RPL13A & digital PCR)				
**Combination**	**0.914 [0.842–0.986]**	**0.437**	**92.3 [79.1–98.4]**	**60.0 [36.1–80.9]**

Abbreviations: 95% CI, 95% confidence interval; AUC, area under the ROC curve.

## 4 Discussion

This case–control study was designed to pair ischemic stroke (IS) patients with healthy controls (HC) based on age, sex, and cardiovascular risk factors assessed using the European Low‐Risk Chart [15]. Clinical comparisons confirmed that IS and HC groups were well matched, minimizing potential bias in RNA expression analyses. Similarly, the ischemic and intracerebral hemorrhage (ICH) groups were comparable with respect to demographics and vascular risk profiles, supporting valid intergroup comparisons. Although the initial NIHSS score was higher in IS than in ICH, three‐month outcomes on the modified Rankin Scale (mRS) were worse in ICH, consistent with the known higher mortality in hemorrhagic stroke. All molecular analyses were performed in triplicate using quantitative PCR and digital droplet PCR (ddPCR), with two validated housekeeping genes yielding consistent normalization results, further supporting the robustness of the data. Blood RNA levels, however, may be influenced by clinical factors including stroke severity, systemic inflammatory state, intercurrent infection, medications, and comorbidities. While our prospective design standardized sampling and processing, the pilot cohort size limited multivariable adjustment.

Using genes previously identified in a primate stroke model [13], we demonstrated that six transcripts—**ADM, DUSP1, HMOX1, HSPA1B, LDLR, and PTGS2**—were significantly upregulated in IS compared with controls. Among the remaining genes, **BAG3** was downregulated, whereas **G0S2** and **TM4SF1** did not differ significantly. These findings extend prior microarray data obtained in peripheral blood mononuclear cells from ischemic stroke patients sampled later after onset (mean 32–53 h) [23]. Our results add that early (< 6 h) overexpression of ADM and DUSP1 can be detected in whole blood, emphasizing their potential utility for ultra‐early diagnosis. To our knowledge, this is the first report describing overexpression of **HMOX1, LDLR, and PTGS2**, and underexpression of **BAG3**, in whole blood after ischemic stroke.

Similar transcriptomic patterns were observed in ICH compared to controls, with early upregulation of **ADM, DUSP1, HMOX1, LDLR**, and **PTGS2**. Elevated circulating adrenomedullin (ADM) protein levels have previously been reported as diagnostic and prognostic markers for ICH [24], consistent with the present RNA‐level findings. Given that cerebral endothelial cells are a major source of adrenomedullin [25], its early blood detection may reflect acute neurovascular injury and endothelial activation.

The biological pathways represented by this nine‐gene panel largely align with well‐described responses to acute ischemic and hemorrhagic injury, including hypoxia and oxidative stress signaling, innate immune activation, proteostasis and autophagy, metabolic reprogramming, and endothelial stress. While these processes are not novel per se, their coordinated detection in peripheral blood is a key translational strength rather than a limitation. Acute stroke is characterized by highly conserved, evolutionarily robust injury programs, and biomarkers intended for early diagnosis or prognostication must reliably capture these dominant systemic responses across individuals rather than reflect rare or idiosyncratic molecular events. Our approach therefore emphasizes biological interpretability and cross‐species consistency, linking primate discovery to human validation, rather than de novo pathway discovery. In this context, the observed associations—particularly those involving stress‐adaptation markers such as BAG3—support the concept that outcome is influenced not only by injury severity but also by the systemic capacity to manage cellular stress and inflammation. These findings provide a mechanistically grounded foundation for larger‐scale validation and refinement, including evaluation against stroke mimics and integration with clinical and imaging predictors.

In stroke, ADM/adrenomedullin is best viewed as a neurovascular stress and repair signal. Human studies show increased ADM expression in peripheral blood leukocytes after ischemic stroke, while experimental work suggests ADM can be protective, reducing apoptosis and promoting angiogenesis after focal cerebral ischemia [26, 27]. DUSP1 is a stress‐response phosphatase that appears to limit ischemia–reperfusion injury. In stroke models, higher DUSP1 activity reduces mitochondrial fragmentation, dampens NLRP3 inflammasome signaling, and decreases neuronal apoptosis; human transcriptomic studies also place DUSP1 within oxidative‐stress and immune‐response signatures in ischemic stroke [28, 29]. HMOX1/HO‐1 is an oxidative‐stress response gene in stroke whether ischemic or hemorrhagic [30]. Experimental work supports a protective role through HIF‐1alpha stabilization and resistance to ischemic injury [31]. HSPA1B belongs to the inducible HSP70 stress‐response program activated by ischemia. Stroke‐specific studies show Hspa1a/Hspa1b upregulation after ischemia, especially in astrocyte endfeet, and the broader HSP70 stroke literature supports a role in proteostasis, anti‐inflammatory signaling, and cytoprotection [32, 33]. In stroke, LDLR has direct mechanistic relevance beyond lipid transport. Experimental cerebral ischemia/reperfusion studies show that LDLR is downregulated after injury, and LDLR deficiency worsens NLRP3‐mediated neuronal pyroptosis, neuroinflammation, and neurological dysfunction [34]. PTGS2/COX‐2 is an acute inflammatory effector in ischemic stroke. Experimental studies identify Ptgs2 as an important acute‐phase neuroinflammatory gene after cerebral ischemia, and COX‐2 signaling also intersects with reperfusion injury pathways including PGE2‐dependent responses and ferroptosis‐related biology [35]. For G0S2, the stroke‐specific literature is currently limited and mostly peripheral‐blood/transcriptomic. A recent PBMC study in ischemic stroke measured G0S2 together with inflammatory genes and found upregulation of GOS2 in ischemic samples compared to controls [36]. BAG3 overexpression in ischemic stroke models attenuates injury by promoting autophagy and reducing apoptosis, making it a plausible stress‐adaptation and recovery‐related marker [37]. For TM4SF1, as with G0S2, the best current stroke‐specific evidence is transcriptomic/biomarker‐oriented. In a human study of transient cerebral ischemia from carotid clamping during carotid endarterectomy, TM4SF1 changed in peripheral blood as part of an early ischemia‐associated gene set, which supports its interpretation as a vascular/ischemia‐responsive blood marker rather than an established causal mediator [38].

A key observation of this study is that combined expression profiling of the nine genes discriminated stroke (ischemic and hemorrhagic) from controls with an AUC of 0.914 (95% CI, 0.84–0.98). The specificity was 60% compared with more than 90% sensitivity. We chose to prioritize sensitivity when determining the positivity threshold in order to rapidly triage patients diagnosed with stroke. Although we would indeed have hoped for a higher level of specificity, the clinical consequences of this misclassification remain limited, as patients will in any case undergo confirmatory imaging following a positive result before any decision is made regarding continuation of care.

In our study, paired healthy controls were compared to ischemic and hemorrhagic stroke. Controls were matched for cardiovascular risk factors with ischemic stroke. In real life, stroke mimics account for about 25% of stroke suspicion. Stroke mimics have less vascular risk factors, younger age, and female predominance, lower (nearly normal) blood pressure, and no or less severe symptoms compared to ischemic stroke patients [39].

In that setting, our level of accuracy compares favorably with previously reported diagnostic biomarker studies. In clinical practice, 20% of strokes are missed and up to 40% of stroke mimics are initially misdiagnosed [40]. A recent meta‐analysis of 223 studies (3494 patients) reported a pooled AUC of 0.89 for protein blood biomarkers, with sensitivity and specificity of 0.76 and 0.84, respectively [41]. Multi‐marker panels performed better than single markers (AUC 0.91 vs. 0.88) [41]. Similarly, the DETECT study [42] using GFAP in 353 patients achieved an AUC of 0.880 for differentiating ICH from IS and stroke mimics, with a 100% negative predictive value in patients with NIHSS > 6. Our data therefore support the feasibility of a multigene RNA signature as a rapid diagnostic tool for early triage in emergency settings. A larger study will allow us to better estimate AUC for both combined ischemic and hemorrhagic strokes but also separately. Our small sample size resulted in wide confidence intervals for AUC and specificity estimates, as well as a high risk of overfitting in the model combining the 9 genes. It is well established that developing prediction models that combine biomarkers requires large sample sizes to reduce overfitting of model coefficients and the consequent overoptimism in discrimination performance. For instance, combining 9 biomarkers with a stroke prevalence of 80% and targeting an AUC of 0.9 would require recruiting at least 246 patients suspected of stroke to satisfy these requirements [43]. Therefore, larger studies conducted in real‐world clinical settings are needed to provide more accurate estimates of the AUC for combined ischemic and hemorrhagic strokes, as well as for each subtype individually.

### 4.1 Comparison of Ischemic and Hemorrhagic Stroke

Direct comparison of IS and ICH revealed that **ADM** and **DUSP1** expression levels were significantly higher in ICH at specific time points. The observed kinetic differences suggest that early transcriptional responses differ substantially between ischemic and hemorrhagic stroke. These distinct temporal signatures support the biological plausibility of transcriptomic profiling as a tool for early stroke subtype discrimination and highlight the importance of considering time‐from‐onset in biomarker development. Larger cohorts will be required to confirm whether the same genes set can achieve high diagnostic accuracy for IS versus ICH discrimination.

### 4.2 Association With Clinical Severity and Prognosis

In IS, initial mRNA expression levels correlated with clinical severity (NIHSS and Glasgow Coma Scale) for **ADM, DUSP1, HMOX1, PTGS2**, and **G0S2**, suggesting that higher expression reflects more extensive injury or systemic response. Early (< 6 h) overexpression of **DUSP1, HMOX1**, and **PTGS2** predicted worse 3‐month outcomes (mRS 3–6). These findings align with prior work showing that ADM expression increases within the first day after ischemic stroke, remains elevated for up to 3 weeks, and correlates with NIHSS, modified Barthel Index, and mRS [26]. For HMOX1, clinical data suggest that higher baseline serum HO‐1 at protein level is however associated with better 3‐month outcome after acute ischemic stroke [44]. For **PTGS2** (also known as COX‐2), although mRNA overexpression has not been previously reported, functional COX‐2 polymorphisms (rs20417, rs5275) have been associated with stroke outcome in a large case–control study [45]. In contrast, no relationship between mRNA expression and clinical severity or outcome was observed in ICH, possibly reflecting different pathophysiological mechanisms or small sample size. Finally, **TM4SF1**, previously proposed in experimental studies [13], did not emerge as a useful biomarker in either stroke type.

### 4.3 Translational Potential and Point‐of‐Care Perspectives

Before clinical implementation, the panel requires (1) multicenter validation in larger cohorts with broader case mix; (2) evaluation against real‐world diagnostic comparators including stroke mimics (e.g., seizure, migraine, functional disorders), CNS infection/inflammation, and other acute systemic illnesses; (3) assessment of incremental value beyond standard clinical and imaging variables using prespecified metrics (e.g., change in AUC, calibration, decision‐curve analysis); and (4) feasibility studies defining turnaround time, analytic robustness, and performance in prehospital or low‐resource environments. If validated point‐of‐care (PoC) molecular technologies could translate these findings into clinical tools for rapid diagnosis. An ideal PoC assay is portable, low‐cost, robust, and delivers results within minutes. While PoC tests for proteins already exist, nucleic acid amplification technologies such as loop‐mediated isothermal amplification (LAMP) now enable RNA detection at the bedside. The RT‐LAMP approach, validated during the COVID‐19 pandemic, can detect RNA targets in under 30 min [46, 47]. Given that RNA changes precede protein expression, mRNA‐based PoC assays might provide earlier diagnostic information than protein‐based platforms.

## 5 Conclusion

In this prospective single‐center pilot cohort, a preselected nine‐gene blood RNA panel demonstrated preliminary ability to distinguish stroke from healthy controls and showed exploratory associations with 3‐month functional outcome. These findings support feasibility and motivate larger multicenter studies—particularly including stroke mimics—to establish clinical utility and generalizability.

Combining multiple biomarkers has already been shown to enhance prognostic accuracy in stroke [48]; Integrating this RNA panel with established clinical and protein markers Could further refine risk stratification for poor 3‐month outcomes. Early identification of patients at risk of long‐term disability would facilitate personalized management strategies aimed at minimizing post‐stroke disability [49].

## Author Contributions

S.R. contributed to the analysis of the data and drafted a significant portion of the final manuscript and figures and reviewed the manuscript. M.B., I.V.‐D., F.‐M.M., D.M., A.J., P.G., and J.C. participated to the acquisition and analysis of data, J.A. and M.C. contributed to the analysis of data and reviewed the manuscript. G.L.G. contributed to the acquisition and analysis of data and reviewed the manuscript. S.T. contributed to the conception and design of the study, the acquisition and analysis of data, drafted a significant portion of the manuscript and figures, and reviewed the manuscript.

## Funding

This work was supported by Société d'Accélération du Transfert de Technologies, 2019_00663_DV_3582.

## Conflicts of Interest

IBIS Study was funded in part by SATT Grand Ouest (Grant #OV: 2019_00663_DV_3582). The authors declare no conflicts of interest. One patent has been filed in relation to this manuscript.

## Supporting information


**Table S1:** Primers and probes sequences used in the study.
**Figure S1:** Evaluation of RT‐qPCR efficiencies of the 8 primers pairs targeting genes used in this study.
**Figure S2:** Kinetic of mRNA relative expression of 6 identified genes in ischemic and hemorrhagic stroke.
**Figure S3:** Time course of mRNA relative expression of 6 identified genes in ischemic stroke with a SMC2 housekeeping gene.
**Figure S4:** Comparative time course of mRNA relative expression of 6 identified genes in ischemic and hemorrhagic stroke with a SMC2 housekeeping gene.
**Figure S5:** Relative mRNA expression of 6 identified genes according to initial clinical severity in ischemic and hemorrhagic stroke with a SMC2 housekeeping gene.
**Figure S6:** Relative mRNA expression of 6 identified genes according to 3‐months clinical handicap in ischemic and hemorrhagic stroke with a SMC2 housekeeping gene.
**Figure S7:** Summary of genes expression according 2 housekeeping genes across time and correlation with NIHSS scores and Rankin scale.
**Table S2:** Initial genes expression and correlation with NIHSS and 3‐months mRS in IS and ICH.
