# Supplementary material for: Blood RNA Biomarker Signatures for Early Diagnosis and Prognosis in Ischemic and Hemorrhagic Stroke: The IBIS‐CT1 Study
Source: Ann Clin Transl Neurol. 2026 Jul 8:10.1002/acn3.70466. Online ahead of print. doi: 10.1002/acn3.70466 (PMC13394127; doi:10.1002/acn3.70466)
Supplement: Supplementary file 1 — Table S1: Primers and probes sequences used in the study. Figure S1: Evaluation of RT‐qPCR efficiencies of the 8 primers pairs targeting genes used in this study. Figure S2: Kinetic of mRNA relative expression of 6 identified genes in ischemic and hemorrhagic stroke. Figure S3: Time course of mRNA relative expression of 6 identified genes in ischemic stroke with a SMC2 housekeeping gene. Figure S4: Comparative time course of mRNA relative expression of 6 identified genes in ischemic and hemorrhagic stroke with a SMC2 housekeeping gene. Figure S5: Relative mRNA expression of 6 identified genes according to initial clinical severity in ischemic and hemorrhagic stroke with a SMC2 housekeeping gene. Figure S6: Relative mRNA expression of 6 identified genes according to 3‐months clinical handicap in ischemic and hemorrhagic stroke with a SMC2 housekeeping gene. Figure S7: Summary of genes expression according 2 housekeeping genes across time and correlation with NIHSS scores and Rankin scale. Table S2: Initial genes expression and correlation with NIHSS and 3‐months mRS in IS and ICH. [file ACN3-9999-0-s001.docx]

**Supplementary Tables and Figures**

**Figure I: Evaluation of RT-qPCR efficiencies of the 8 primers pairs targeting genes used in this study.**

**Table I: Primers and probes sequences used**


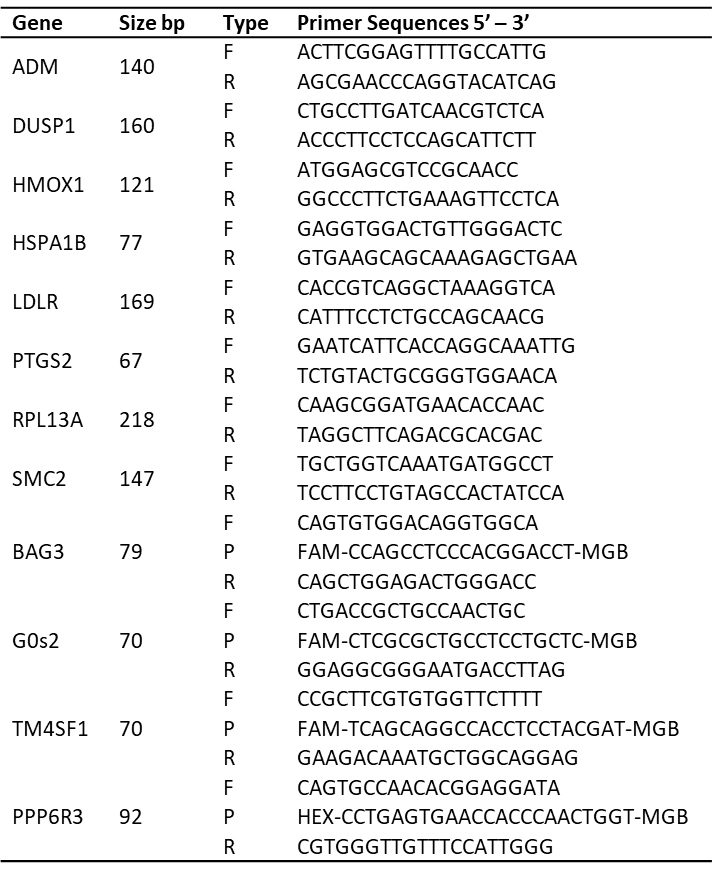

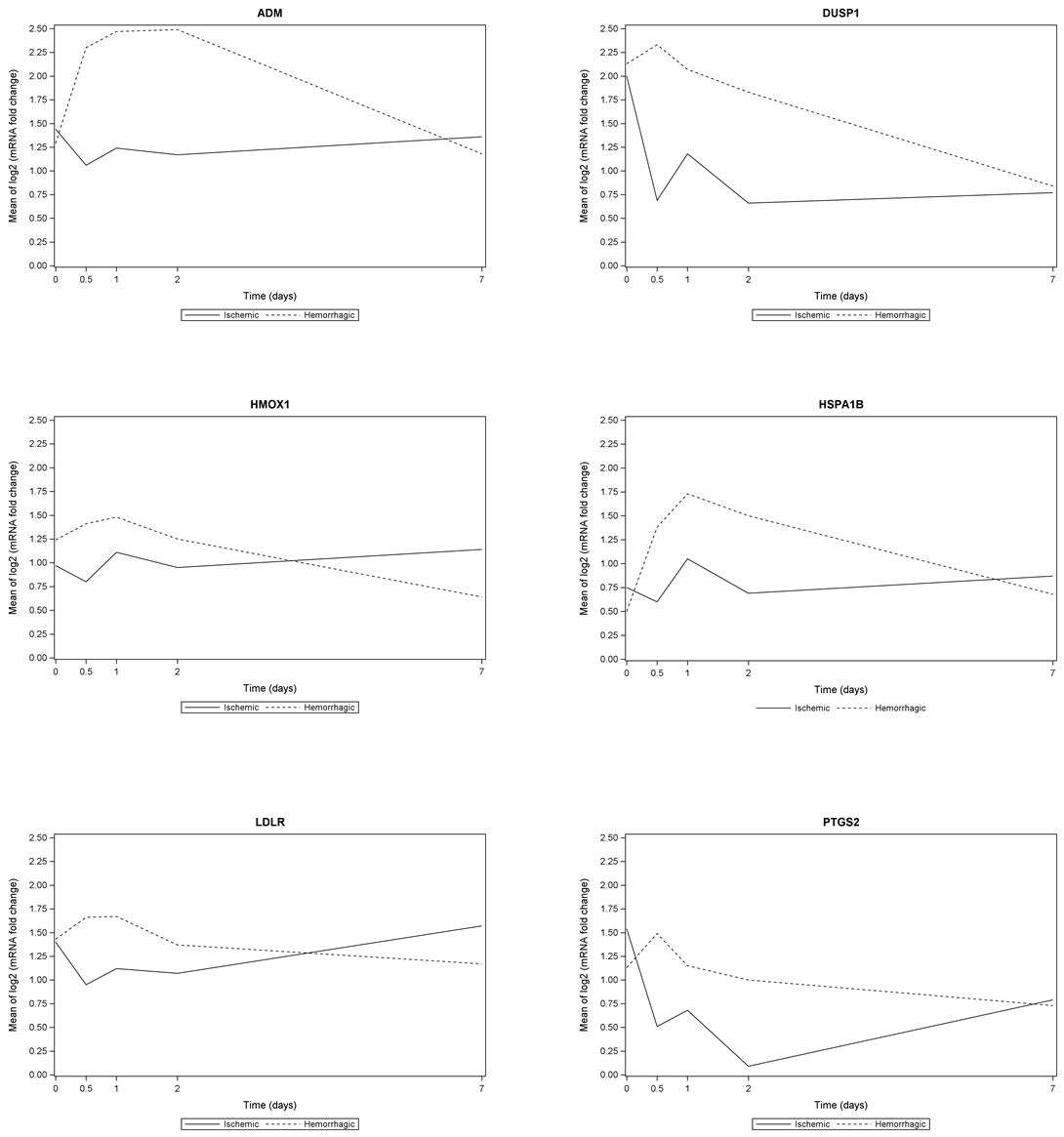


**Figure II: Kinetic of mRNA relative expression of 6 identified genes in ischemic and hemorrhagic stroke**

**Figure III. Time course of mRNA relative expression of 6 identified genes in ischemic and hemorrhagic stroke**

**with housekeeping gene SMC2.**

**B**

**A**

**A**

**B**

**Figure V: Relative Expression of 6 identified genes according to clinical severity in ischemic and hemorrhagic stroke**

**Figure IV. Comparative time course of mRNA relative expression of 6 identified genes in ischemic and hemorrhagic stroke.**

**B**

**A**

**Figure VI: Relative Expression of 6 identified genes according to 3-months clinical handicap in ischemic and hemorrhagic stroke.**


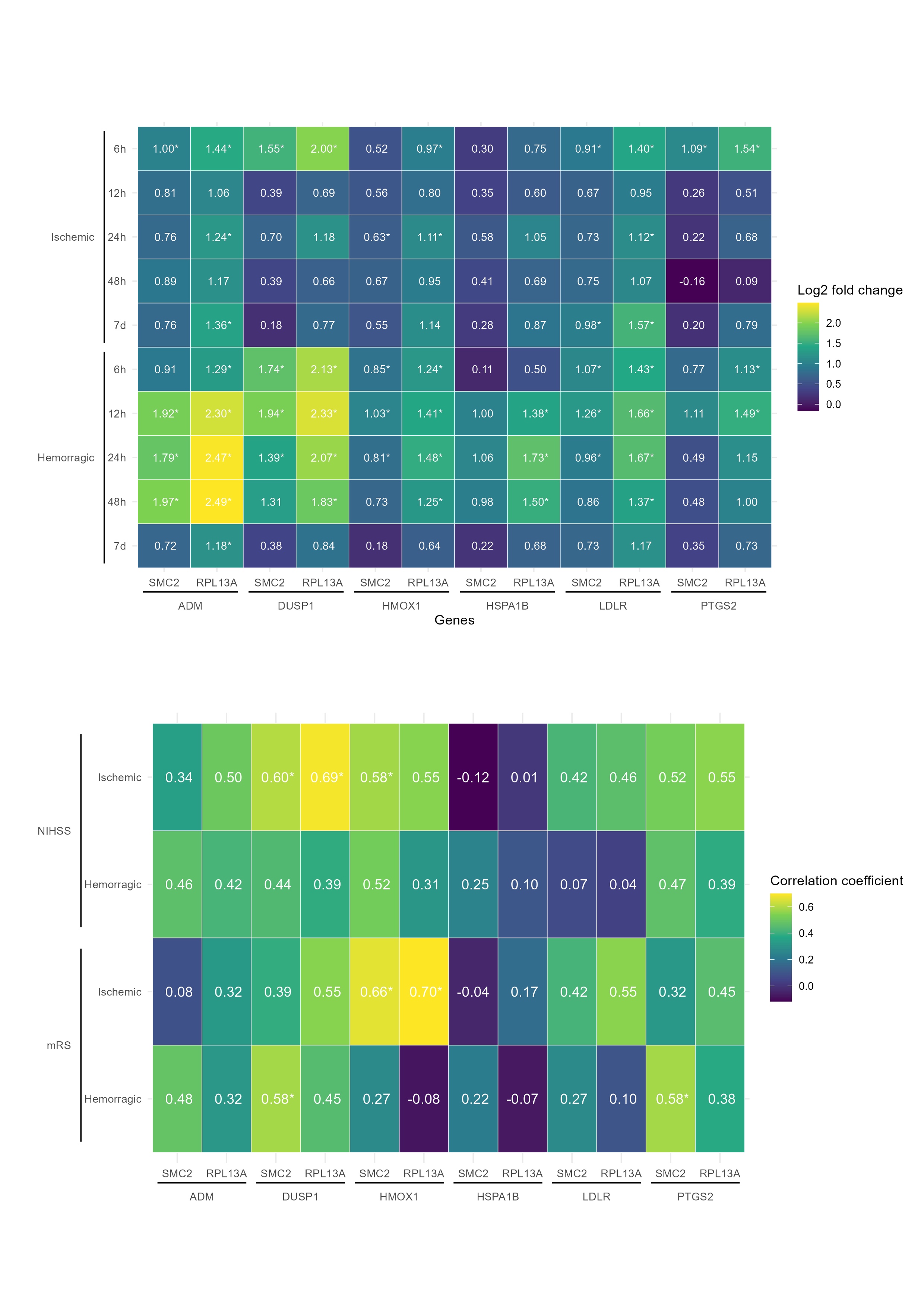


**Figure VII: Genes expression summary according 2 housekeeping genes across time and correlation with NIHSS and Rankin scale**

**Supplementary Table 2: Genes expression and correlation with scores in IS and ICH at patients admission**

**A**

| **Reference**  **gene** | **Stroke**  **types** | **Mean log2(mRNA FC) ± SD at <6h from qPCR data** | | | | | | **from ddPCR data** | | |
| --- | --- | --- | --- | --- | --- | --- | --- | --- | --- | --- |
|  |  | **ADM** | **DUSP1** | **HMOX1** | **HSPA1B** | **LDLR** | **PTGS2** | **BAG3** | **G0s2** | **TM4SF1** |
| **RPL13A (qPCR)**  **or**  **PPP6R3 (ddPCR)** | IS | 1,44±1,19  p=0,0001 | 2,00±1,52  p=0,0001 | 0,97±1,19  p=0,0016 | 0,75±1,20  p=0,0118 | 1,40±1,37  p=0,0002 | 1,54±1,44  p=0,0001 | - 0,96±0,79  p=0,0001 | 0,55±1,66  p=0,2611 | 0,24±1,51  p=0,4958 |
|  | ICH | 1,29±1,46  p=0,0012 | 2,13±1,84  p=0,0001 | 1,24±1,05  p=0,0001 | 0,50±1,17  p=0,0812 | 1,43±1,13  p=0,0001 | 1,13±1,36  p=0,0020 | - 0,75±0,95  p=0,0323 | 0,73±2,49  p=0,2798 | 0,13±0,98  p=0,9537 |
| **SMC2** | IS | 1,00±1,32  p=0,0031 | 1,55±1,49  p=0,0002 | 0,52±0,86  p=0,0161 | 0,30±1,13  p=0,2469 | 0,91±1,06  p=0,0012 | 1,09±1,26  p=0,0010 | NA | NA | NA |
|  | ICH | 0,91±1,67  p=0,0298 | 1,74±2,00  p=0,0013 | 0,85±0,96  p=0,0011 | 0,11±1,15  p=0,6767 | 1,07±1,11  p=0,0027 | 0,77±1,49  p=0,0364 | NA | NA | NA |

**B**

| **Reference**  **gene** | **Stroke**  **types** | **Correlation of NIHSS score with gene expression at <6h from qPCR data** | | | | | | **from ddPCR data** | | |
| --- | --- | --- | --- | --- | --- | --- | --- | --- | --- | --- |
|  |  | **ADM** | **DUSP1** | **HMOX1** | **HSPA1B** | **LDLR** | **PTGS2** | **BAG3** | **G0s2** | **TM4SF1** |
| **RPL13A (qPCR)**  **or**  **PPP6R3 (ddPCR)** | IS | r=0,50  p=0,0259 | r=0,69  p=0,0008 | r=0,55  p=0,0121 | r=0,01  p=0,9795 | r=0,46  p=0,0416 | r=0,55  p=0,0114 | r= - 0,43  p=0,0581 | r=0,62  p=0,0039 | r= - 0,11  p=0,8368 |
|  | ICH | r=0,42  p=0,0725 | r=0,39  p=0,1020 | r=0,31  p=0,1927 | r=0,10  p=0,6692 | r=0,04  p=0,8852 | r=0,39  p=0,1032 | r= - 0,28  p=0,2484 | r=0,14  p=0,5802 | r= - 0,21  p=0,3919 |
| **SMC2** | IS | r=0,34  p=0,1404 | r=0,60  p=0,0048 | r=0,58  p=0,0073 | r= - 0,12  p=0,6214 | r=0,42  p=0,0629 | r=0,52  p=0,0191 | NA | NA | NA |
|  | ICH | r=0,46  p=0,0451 | r=0,44  p=0,0610 | r=0,51  p=0,0240 | r=0,25  p=0,3040 | r=0,07  p=0,7884 | r=0,47  p=0,0430 | NA | NA | NA |

**C**

| **Reference**  **gene** | **Stroke**  **types** | **Correlation of 3M mRS with gene expression at <6h from qPCR data** | | | | | | **from ddPCR data** | | |
| --- | --- | --- | --- | --- | --- | --- | --- | --- | --- | --- |
|  |  | **ADM** | **DUSP1** | **HMOX1** | **HSPA1B** | **LDLR** | **PTGS2** | **BAG3** | **G0s2** | **TM4SF1** |
| **RPL13A (qPCR)**  **or**  **PPP6R3 (ddPCR)** | IS | r=0,32  p=0,1785 | r=0,55  p=0,0148 | r=0,70  p=0,0009 | r=0,17  p=0,4783 | r=0,55  p=0,0142 | r=0,45  p=0,0515 | r= - 0,18  p=0,4483 | r=0,45  p=0,0525 | r= - 0,03  p=0,9029 |
|  | ICH | r=0,32  p=0,1869 | r=0,45  p=0,0552 | r= - 0,08  p=0,7397 | r= - 0,07  p=0,7662 | r=0,10  p=0,6857 | r=0,38  p=0,1104 | r= - 0,40  p=0,0932 | r=0,36  p=0,1310 | r=0,01  p=0,9696 |
| **SMC2** | IS | r=0,08  p=0,7301 | r=0,39  p=0,0993 | r=0,66  p=0,0019 | r=0,04  p=0,8782 | r=0,42  p=0,0748 | r=0,32  p=0,1840 | NA | NA | NA |
|  | ICH | r=0,48  p=0,0373 | r=0,58  p=0,0088 | r=0,27  p=0,2636 | r=0,22  p=0,3549 | r=0,27  p=0,2558 | r=0,58  p=0,0089 | NA | NA | NA |
